# Welcome to *Animal Microbiome*

**DOI:** 10.1186/s42523-019-0003-5

**Published:** 2019-02-05

**Authors:** Sharon A. Huws

**Affiliations:** School of Biological Sciences, Queen’s University Belfast, University Road, Belfast, Northern Ireland BT7 1NN UK

I am proud to announce the official launch of *Animal Microbiome*. We launch today with ‘Distinct microbiotas of anatomical gut regions display idiosyncratic seasonal variation in an avian folivore [1]’ and have many articles accepted and ready for publication in due course.

So you may ask why do we need yet another journal with a focus on microbiomes? When I was approached regarding my thoughts on the need for an animal microbiome focussed journal, I asked myself the same question: is it really needed? The rapid rise in microbiome research (defined by Marchesi and Ravel [2] as the entire habitat, including the microorganisms, their genomes (i.e., genes), and the surrounding environmental conditions) in recent years, due to the explosion in sequencing technologies, has led to an upsurge in published articles that use ‘omic approaches to understand the complexities of microbiomes. In order to accommodate this increase, the journal *Microbiome* was launched in 2013. This journal has gone from strength to strength with the current impact factor being 9.133. Indeed, for scientists focussed on microbiomes, this journal has become the go-to place to publish due to its reputation. This is excellent, but has led to a strain on capacity to deal with a high influx of submissions. The journal was also originally set up to focus on clinical and environmental microbiomes, but in recent years there has been a continual increase in the number of animal microbiome related publications. As a Senior Editor of *Microbiome* in the animal section, I can say that we are inundated with excellent submissions but often they may not have quite the impact required for *Microbiome*. So where do you submit to if your paper is rejected from *Microbiome*? There is, of course, an array of journal options, but there is a gap for an impactful journal focusing solely on animal microbiome studies, and this is the main impetus for launching the journal *Animal Microbiome*.

Being a sister journal to *Microbiome, Animal Microbiome* will have the same high standards of openness and transparency. To facilitate the increase in animal microbiome submissions to *Microbiome* and the launch of *Animal Microbiome*, we have increased the pool of editors, thus aiding speed of processing. This pool includes editors with expertise in an array of different animal-focussed microbiomes (e.g., those associated with ruminants, poultry, domestic animals).

*Animal Microbiome* will address all aspects of non-human animal-associated microbiomes, including (but not limited to): domestic, wild and livestock animals. We will also welcome studies encompassing (but not limited to): marker gene surveys; ‘-omics’ surveys (including culturomics, metagenomic, metatransciptomic, metaproteomic, and metabolomic), bioinformatic and other analytical tools, which have an underlying strong hypothesis for using such techniques. The use of meta-omic approaches is strongly encouraged in order to substantially advance scientific understanding. *Animal microbiome* operates under the same principles and policies as *Microbiome*. Once accepted, all articles published in *Animal Microbiome* will be freely available through the journal web portal and within major research publication databases such as PubMed Central in due course. Also, because authors, not the publisher, will hold the copyright to their work, they will be free to distribute published articles. Open access science is crucial for ensuring the most impact from published research.

The number of submissions we have already had bodes well for the need for this journal and the future of the journal. As the Editor-in-Chief for *Animal Microbiome,* my strategy is for the journal to become a platform for understanding animal-associated microbiomes and allow scientific exchange of ideas to increase the impact of research in this field. We hope that you will support this journal in order to advance fundamental understanding of animal associated microbial communities globally.

## Author’s information

Sharon Huws is the Editor-in-Chief of Animal *Microbiome* and Senior Editor on *Microbiome*.
